# Carbon stock in Japanese forests has been greatly underestimated

**DOI:** 10.1038/s41598-020-64851-2

**Published:** 2020-05-12

**Authors:** Tomohiro Egusa, Tomo’omi Kumagai, Norihiko Shiraishi

**Affiliations:** 1 Graduate School of Agricultural and Life Sciences, The University of Tokyo, 1-1-1 Yayoi, Bunkyo-ku Tokyo, 113-8657 Japan; 2 Institute for Space-Earth Environmental Research, Nagoya University, Furo-cho, Chikusa-ku Nagoya, 464-8601 Japan

**Keywords:** Biogeochemistry, Environmental sciences

## Abstract

An accurate estimate of total forest carbon (C) stock and C uptake is crucial for predicting global warming scenarios and planning CO_2_ emission reductions. Forest inventory, based on field measurements of individual tree sizes, is considered the most accurate estimation method for forest C stock. Japan’s national forest inventory (NFI) provides stand-scale stem volume for the entire forested area based on (1) direct field measurements (m-NFI) and (2) prediction using yield tables (p-NFI). Here, we show that Japanese national and local forestry agencies and some research studies have used p-NFI and greatly underestimated the Japanese forest C stock (58–64%) and net annual C uptake (41–48%). This was because approximately 10% of the forest area was not counted in p-NFI and because the yield tables in p-NFI, which were constructed around 1970, were outdated. For accurate estimation of the forest C stock, yield tables used in p-NFI should be reconstructed or ideally field measurement campaigns for m-NFI should be continued. In the future, appropriate forest management plans are necessary to effectively use the high CO_2_ absorption capacity of Japanese forests and these should be compared with other industries’ CO_2_ reduction plans from a cost-benefit perspective.

## Introduction

Forests accumulate large amounts of Carbon (C) from the atmosphere by incorporating photosynthesis into long living woody biomass^1,2^, thereby greatly affecting the rate of increase of atmospheric CO_2_ concentration and global warming^3^. The Marrakesh Accords determined that net C uptake, which reflects the temporal variation in forest C stocks, can be counted in national CO_2_ reduction plans if the forest is properly managed^4^; this ruling remains active^5^. Therefore, an accurate estimate of the total forest C stock and net C uptake at national to global scales is crucial for predicting future global warming scenarios and planning future CO_2_ emission reductions.

The national forest inventory (NFI) is a national forest survey that was historically aimed to estimate timber productivity. It generally includes information on tree species composition and stand-scale or individual tree-scale stem/timber volume in targeted forests. The Nordic countries began using the NFI from the 1910s^6^. Although each country has its own survey standards^7,8^, the NFI generally provides an accurate and valid estimation of national forest C stocks and net C uptake because it is based on multipoint observations and the uncertainties of the estimated values can be calculated^9–11^. Using NFI data, many studies have clarified the forest C stocks and net C uptake from the past to the present^6,9,11–16^ and predicted their future changes with forest growth models^17–19^.

**Figure 1:**
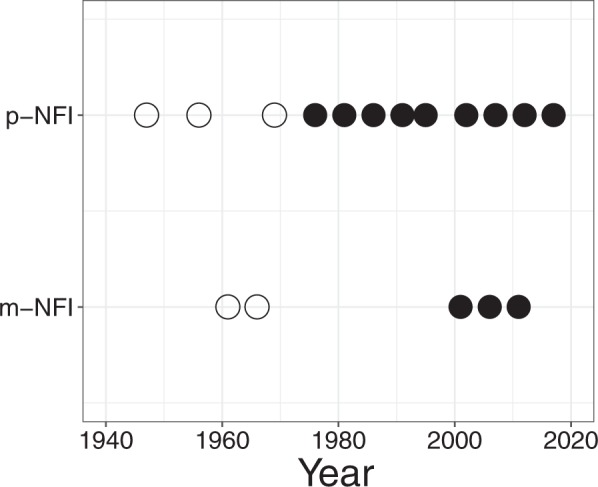
Survey years of National Forest Inventory (NFI) in Japan. p-NFI and m-NFI denote NFI based on the predictions from yield tables and the direct field measurements, respectively. Open circles mean that the surveys were done with different methods to the current ones. Each m-NFI survey has a 5-year field measurement period; therefore, m-NFIs that began in 1999 are plotted at their central years.

In Japan, two types of NFI have existed (Fig. 1). One (hereinafter referred to as p-NFI) is based on predictions from yield tables, which are empirical relationships between stand age and the stand stem volume. These yield tables were constructed using actual observation data obtained around 1970. The other is based on direct field measurements (hereinafter referred to as m-NFI). Using 10,000 survey points m-NFI surveys were carried out in 1961, 1966 and, following a long gap, 1999, based on revised sampling methods^20^ (Fig. 1). Despite its broader use, p-NFI tends to underestimate the actual stem volume^21^. The m-NFI conducted in 1999–2003 was also observed to underestimate the stem volume^22^. However, for the m-NFI in 2009–2013, experts checked the accuracy and surveyors underwent technical training. Thus, the m-NFI in 2009–2013 was the most reliable NFI in Japan.

In fact, few studies with scientific accuracy and of academic value have evaluated Japan’s nationwide C stock. In this study, we assumed that even the few studies recognized as academically valid have committed a fallacy: some studies estimated the C stock using p-NFI, which resulted in an apparent underestimation^23,24^, and some examined the climate and environmental change effects on C uptake by mistaking p-NFI for m-NFI, which caused them to misunderstand the effect^25–28^. These studies might have misled policymakers in their Japanese forest management policy and legislation. Hence, our main objective in this study was to offer an accurate estimation of Japan’s forest C stock. To achieve this, using the latest m-NFI estimations, we clarified the extent to which p-NFI underestimates the actual values and rectified the long-term net C uptake.

## Results and Discussion

**Figure 2:**
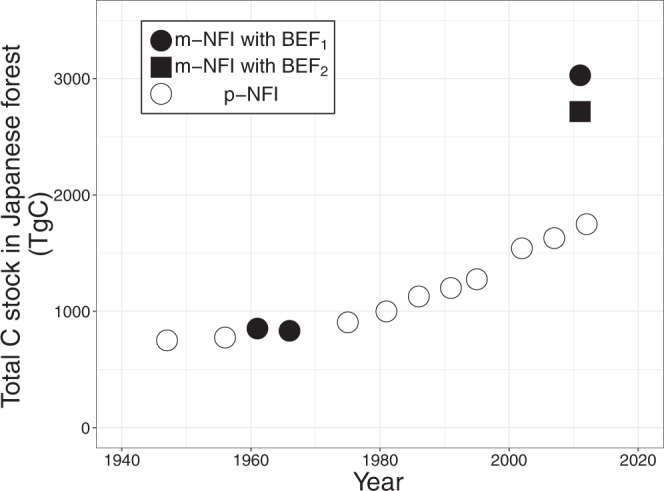
Temporal changes in total forest carbon stock in Japan. p-NFI and m-NFI denote National Forest Inventory (NFI) based on the predictions from yield tables and the direct field measurements, respectively. The carbon stocks until 1995 were estimated by Fang *et al*. (2005)^25^. BEF_1_ and BEF_2_ represent two different biomass expansion factor (BEF) types. BEF_1_ and BEF_2_ were used for all calculations and a calculation from m-NFI in 2009–2013, respectively (see Methods section). We did not show the 95% confidence interval because it was invisibly small.

C stocks estimated from m-NFI in 1961 and 1966 were 851.7 ± 23.0 (the plus-minus sign represents the 95% confidence interval) and 834.0 ± 22.5 TgC, respectively, which lie between values from p-NFI in 1956 (774.2 TgC) and 1975 (905.8 TgC) (Fig. 2). Until the 1970s, C stocks estimated from p-NFI were expected to be equivalent to those estimated from m-NFI, because the yield table for each p-NFI was constructed using the field data at that time^23^. Thus, we can confirm that the total forest C stocks from the 1940s to the 1970s were not more than 1000 TgC and were somewhat constant (that is, the annual net C uptakes were small).

The m-NFI-derived C stock in 2011 was estimated using two types of BEF (BEF_1_ and BEF_2_; see the Methods) to give 3016.2 ± 26.9 and 2696.4 ± 25.4 TgC, respectively (Fig. 2). The p-NFI-derived C stock in 2012 was 1750.0 TgC, which was considerably smaller than the m-NFI values. Additionally, considering the validation surveys since 2010, the m-NFI probably underestimates the stem volume by 4–8%^29,30^. This suggested that the difference between the p-NFI- and m-NFI-derived C stocks would be even greater than the present study showed. Therefore, we can conclude that the p-NFI-derived C stock greatly underestimated the actual forest C stock in Japan.

Such a discrepancy between p-NFI- and m-NFI-derived C stocks resulted in a large difference in the average net C uptakes between p-NFI and m-NFI: 19.9 TgC year^−1^ for 1966–2012 using p-NFI and 48.5 ± 0.8 and 41.4 ± 0.8 TgC year^−1^ for 1966–2011 using m-NFI with BEF_1_ and BEF_2_, respectively. The m-NFI-derived net C uptake was more than twice that of the p-NFI value. The area-based m-NFI C uptakes with BEF_1_ and BEF_2_ were 1.83 ± 0.03 and 1.56 ± 0.03 MgC ha^−1^ year^−1^, respectively, and that of p-NFI was 0.84 MgC ha^−1^ year^−1^ (Here, we assumed the forest area in 1966 was equal to that in 2012 p-NFI, 23.7 million ha). The net ecosystem productivity (NEP) observed using a micrometeorological method (the eddy-flux measurement) at seven forest sites across Japan ranged from 1.23 to 3.88 MgC ha^−1^ year^−1^ ^31^. The NEP estimated using a carbon cycle model and eddy-flux data was more than 1 MgC ha^−1^ year^−1^ in most areas of Japan^32^. Additionally, the NEP of Japanese deciduous broadleaf forest estimated from MODIS sensor and eddy-flux data was 3.47 ± 2.88 MgC ha^−1^ year^−1 ^^33^. These NEP values were comparable with the net C uptakes estimated from m-NFI, suggesting the validity of m-NFI-derived net C uptake and C stock.

**Figure 3:**
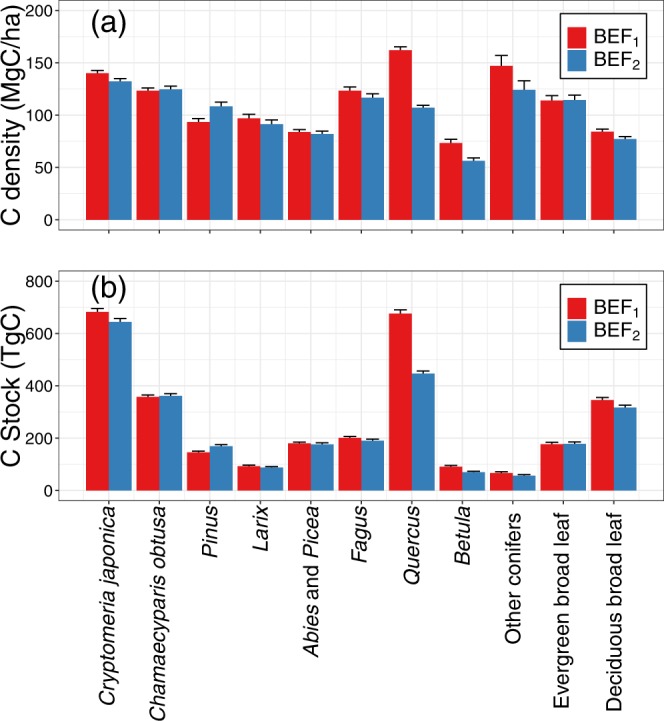
(**a**) Carbon density and (**b**) carbon stock for each forest type estimated using National Forest Inventory data based on the direct field measurements (m-NFI) in 2009–2013. BEF_1_ and BEF_2_ represent two different biomass expansion factor (BEF) types (see Methods section). Error bars represent the upper limits of 95% confidence interval.

We also estimated C stock and the area-based C stock (C density) for major forest types in Japan using m-NFI with BEF_1_ and BEF_2_ (Fig. 3). *Cryptomeria japonica*, *Chamaecyparis obtusa*, *Fagus* and *Quercus* have almost the same level of C density (ca. 120 MgC ha^−1^), which implies their relatively higher productivity (Fig. 3a). *Cr. japonica* and *Quercus* (famil) in Japan had the dominant C stocks in those forest types (Fig. 3b). In terms of an estimation error caused by the adoption of BEF_1_ or BEF_2_, we found that the BEF_1_ estimations were greatly larger than the BEF_2_ ones for *Cr. japonica* (5.6%), *Lalix* (5.8%), *Fagus* (5.5%), *Quercus* (33.9%), other conifers (15.6%) and deciduous broadleaf trees (8.3%), and greatly smaller for *Pinus* (16.1%; Fig. 3). Due to the difference of the C content in the calculations with BEF_1_ and BEF_2_ (Eqs. 2 and 3), the C stocks with BEF_2_ were 2% higher and 4% lower than those with BEF_1_ for conifers and broadleaf forests, respectively. Even considering the effects of the C contents, there were still large differences between the C stocks estimated with BEF_1_ and BEF_2_ in some forest types. In particular, the C stocks of *Quercus* using BEF_1_ and BEF_2_ were estimated as 676.1 ± 14.1 and 447.1 ± 9.7 TgC, respectively (Fig. 3b). The difference between the two accounted for most of the difference between the total forest C stocks estimated with BEF_1_ and BEF_2_ (ca. 300 TgC; Fig. 2). Because the C stock of *Quercus* seems comparable to that of *Cr. japonica*, successfully adopting the BEF method for *Quercus* might be critical for the estimating Japan’s total forest C stock.

**Figure 4:**
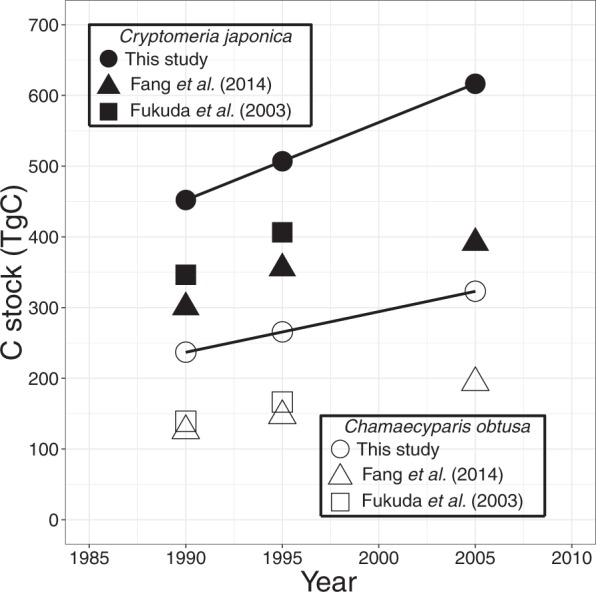
Temporal change in carbon stock for *Cryptomeria japonica* and *Chamaecyparis obtusa* in 1990–2005, estimated using m-NFI with BEF_1_ (circles: see Methods section), by Fang *et al*. (2014)^28^ (triangles) and by Fukuda *et al*. (2003)^23^ (squares).

Only two tree species, *Cr. japonica* and *Ch. obtusa*, the major plantation tree species for timber production in Japan, comprise 34.5% of Japan’s total forest C stock (Fig. 3). We estimated the *Cr. japonica* and *Ch. obtusa* C stocks in 1990, 1995 and 2005 using m-NFI with BEF_1_ (see Methods section) and compared to those in two previous studies^23,28^. One previous study used a simple growth function with parameters derived from raw data for national stand density diagrams in the 1970s^23^ and the other simply used p-NFI^28^. The estimated C stocks were 452.1, 507.0 and 616.7 TgC for *Cr. japonica* and 236.8, 265.5 and 323.0 TgC for *Ch. obtusa*, in 1990, 1995 and 2005, respectively, which were much higher than those in the previous studies (Fig. 4). Again, these results demonstrated that the past C stock calculations with p-NFI have greatly underestimated the actual forest C stock.

There are several possible factors for the large discrepancy between p-NFI and m-NFI. One potential factor is the difference in forested areas measured in p-NFI and m-NFI. Although the area categorized as forest in m-NFI in 2009–2013 was 25.9 million ha, the total area of planted and natural forests in p-NFI was 23.7 million ha in 2012^34^. This difference results in a C stock underestimation of approximately 10%. In p-NFI, 1.6 million ha of forested area was reported as the sum of treeless land and bamboo forest, which would be a major cause of the difference (ca. 2.2 million ha) between p-NFI and m-NFI. The majority of this 1.6 million ha was treeless land, because bamboo forest is estimated to occupy only 1% of the total forest area (~0.3 million ha; See Methods section). Fundamentally, when harvesting or planting forests in Japan, submitting a report to the local government is required. If no notification of planting is made after harvesting, the area will be treated as treeless land. It is quite possible there exist forests in some treeless land because of the natural growth of trees. Additionally, the forested area may have increased slightly because of conversion from agricultural land to forest. Only if the owner reported the change, the area could be reflected in p-NFI.

Another potential factor is that the yield tables of p-NFI, which had been constructed around 1970, were outdated with the following reasons.

1. The underestimation of older forests’ growth. There were few older forests in Japan around 1970 because many forests were clear-cut after World War II to meet increased timber demand^35^. Furthermore, the remaining older forests at that time might have been so poor growth that they were not worth harvesting. Thus, older forests’ growth characteristics were not reflected in the yield tables, which caused C stock underestimation for the older forests and the total forests. Although the latest studies found a higher growth rate than expected in older forests^36,37^, few studies on older forests’ growth have been conducted in Japan^38,39^. Therefore, intensive observations of older forests should be performed in the future and reflected in the yield tables.
2. The increased forest growth due to changes in environmental factors. Many experimental studies have shown that rising air temperatures can enhance vegetation growth^40,41^. Besides, the net primary production of Japanese forests was strongly constrained by air temperature and solar radiation^31,42,43^. Therefore, it is highly probable that the rate of Japanese forest growth increased with the recent rise in air temperature. Furthermore, Nitrogen (N) deposition has increased in Japan since the 1980s because of emissions from China^44^. Of the non-N limitation conditions, increasing atmospheric CO_2_ concentration possibly enhanced forest productivity^45–48^.
3. The lack of thinning. A main purpose of the yield table is to predict stem/timber production volume for a target commercial tree species on condition that common forestry practices are conducted in a given environment. However, a decline in timber prices in Japan over many years has reduced the motivation of forestry practices, and in particular, the lack of thinning has become a serious problem^35,49^. Some previous studies reported that the stand-level forest stock of non-thinned plots was comparable with^50^ or significantly higher than that of thinned plots^50–52^, even decades after thinning. Therefore, the lack of thinning in Japanese forests might have led to an increase in forest C stock.

## Concluding Remarks

Our study shows that p-NFI, which Japanese public forestry agencies have believed to be authentic, has underestimated the m-NFI-derived actual total C stock in Japanese forests. The main causes are the underestimation of the forest area of 2.2 million ha and the underestimation of the forest biomass due to the outdated yield tables. Our results have implied that reconstructing the yield tables in p-NFI and ideally changing the timber production estimation system completely from p-NFI to m-NFI is necessary for accurate estimation of forest C stock. In principle, m-NFI is more suitable for accurate estimation of forest C stock and decision-making than p-NFI because m-NFI estimates forest area more accurately than p-NFI and calculates uncertainties of the results. We should encourage Japanese forest management policymakers and legislators to adopt the revised timber production/ forest C stock estimation system.

The present m-NFI system began in 1999; the fourth survey of 2014–2018 is now complete and is currently being analyzed. The accuracy check in m-NFI is ongoing, but the latest result shows an annual mean difference between the actual and estimated stem volume for 2014–2016 of 5–7%^30^, which suggests a successful effort to improve the accuracy of m-NFI estimation. At present, only stand-area-based stem volume data are available in the m-NFI data supply system. Therefore, we applied the stand-area-based BEF for a single dominant tree species in a given stand to the estimation of forest stand C stock, which is a potential source of error. The wider availability of individual tree-scale configuration data such as stem volume, tree height and diameter at breast height (DBH) will allow us to more accurately estimate forest stand C stock. Furthermore, these data can describe the status of Japanese forests in detail, revealing the factors that yield tables underestimating current forest stocks. Elucidation of these factors is one possible method for accurately correcting past Japanese forest C stocks and predicting future changes. It could help policy makers in their decision-making, and therefore, is an important future task.

As previous studies noted^53^, BEF use can cause errors when estimating the forest C stock (Figs. 2 and 3). In particular, we found a significant difference in C stock estimation between BEF_1_ and BEF_2_ for the *Quercus* family, which is a major forest type and has significant biomass in Japan (Fig. 3). Again, the non-availability of raw data is a major obstacle. Integrating the data used for BEF_1_ and BEF_2_ would enable us to obtain BEF more accurately.

Biomass and C dynamics in forest ecosystems in warm-cool temperate zones, which have the largest population and the most active human life in the world, have received increasing attention. Therefore, various studies relating to the C uptake process, such as eddy-covariance measurements, remote sensing and terrestrial ecosystem process modelling, have been conducted. We emphasize that forest inventory based on direct field measurements conducted at numerous plots is the strongest tool to validate results from such studies. The present study can help the researchers of these related fields to correctly understand and properly use the current NFI in Japan and will contribute to the progress of C dynamics studies in warm-cool temperate zones.

## Methods

### National forest Inventory based on direct field measurements: m-NFI

In one campaign of the current m-NFI survey, direct field measurements were conducted for 5 years at more than 10,000 plots throughout Japan^20^. These plots formed a 4-km grid interval across Japan. Measurements were conducted at given plots including forests. The third m-NFI survey was conducted in 2009–2013 (Fig. 1); the data can be downloaded from the Japan Forestry Agency website (http://www.rinya.maff.go.jp/j/keikaku/tayouseichousa/). The number of planned plots was initially 14,838; actual forest plots were then selected, which resulted in 14,719 plots (14,522, if eliminating bamboo plots). Practically, however, the number of the target plots was 13,357 because of their accessibility.

Each plot comprises triple concentric circles with areas of 0.01, 0.04 and 0.1 ha. In the largest (0.1 ha), medium (0.04 ha) and smallest (0.01 ha) circular areas, the DBHs of trees ≥18, 5 and 1 cm DBH, respectively, are measured. Tree height is measured for each of the selected 20 trees in the plot and tree heights of the remaining trees are estimated using the DBH–tree height relationships derived from the 20 tree measurements. Stem volume of a single tree is calculated from the empirical relationship between DBH and tree height for each tree species and its growing region^54,55^. Note that the publicly available data do not include individual tree scale information as mentioned above, but rather the plot scale totalized information.

This study used only the published data, that is, stem volume per hectare (*V*s; m^3^ ha^−1^), name of dominant tree species and forest age for a given plot. Here, the dominant tree species denotes a tree species with the largest total cross-section area at breast height in the plot. The total number of plots used for the survey was 12,457 because we excluded 703 plots in which not all of the three types of the data were identified and 197 bamboo plots for which the C stocks were difficult to estimate. Measurement validation commenced in 2010. The annual mean differences in *V*s between the practical measurements and the validation surveys were 4%–8% in 2010–2013^29^.

### National forest inventory based yield table predictions: p-NFI

The yield tables used in p-NFI show empirical relationships among stand-scale stem volume, age class (ranked from 1 at 5-year intervals) and land-productivity index for each major tree species. Although the land-productivity index is critical for estimating the stem volume in a given stand, no clear standard existed for many target forest stands on how the land-productivity index was determined. As far as we investigated, no one in national and local forestry agencies knows how to revise the land-productivity index, which can be affected by various environmental changes, which are a large source of error for the stem volume estimation. Note that the currently-used yield tables were constructed using the actual measurements from around 1970.

Yield tables for Japan differ depending on the type of forest-ownership. For national forests (ca. 30% of the total forested area), yield tables created by the national government have been used. However, for local-government-owned and private forests (ca. 70%), each of 47 prefectural governments to which the given forests belong created its own yield tables. Surprisingly, most yield tables have not been published and even the national forestry agency, that is, the Japan Forestry Agency, cannot know all the information on their yield tables. The Japan Forestry Agency has aggregated the data submitted by administrators for national and prefectural forests and published estimations of the stem volume with some relevant information (http://www.rinya.maff.go.jp/j/keikaku/genkyou/index1.html).

The forests were categorized into two types in p-NFI: plantations and natural forests. Data for plantations comprises stem volume and forest area, which are further categorized into 10 forest types, 47 prefectures and 19 age classes. Data for natural forests include only stem volume categorized into 11 forest types and 47 prefectures. Notably, no information on forest area for each forest type is included for natural forests.

### Carbon density estimation using biomass expansion factor (BEF)

BEF is the coefficient derived from allometry measurements, which enables us to convert *V*s to the stand scale C stock. BEF has several definitions, and one type of BEF calculates total biomass (sum of stems, branches, leaves and roots) and another type calculates aboveground biomass (sum of stems, branches and leaves). Previous studies in Japan have used both types of BEF; here, we refer to these as BEF_1_ and BEF_2_. BEF_1_ was suggested in a study examining long-term changes in Japanese forest C stocks^25^ We used the past forest C stock values that were calculated with BEF_1_ in a previous study^25^; therefore, BEF_1_ is indispensable for the present study. BEF_2_ was used in the official reports from the Japanese government^56^ and is probably a more commonly used BEF in Japan than BEF_1_. Therefore, we estimated all forest C stocks with BEF_1_ to clarify the temporal changes in forest C stock and used both BEFs for m-NFI in 2009–2013 to quantify the uncertainty derived from the difference in BEF. The conversion methods using these BEFs are shown below.

BEF_1_ (Mg m^−3^) is for the conversion from *V*s to total biomass per hectare and was presented for 10 plantation-forest types in p-NFI. According to the evidence that BEF_1_ tends to increase with decreasing *V*s, the following equation is reached:

1 $${{\rm{BEF}}}_{1}={\rm{a}}+b/V{\rm{s}},$$

where a (Mg m^−3^) and b (Mg ha^−1^) are empirical parameters obtained for a given forest type. In the case that *V*s is extremely small in Eq. 1, BEF_1_ becomes unrealistically large. Therefore, we set an upper limit of 1.5 for BEF_1_ according to data obtained in a previous study^25^. In addition, in previous studies using BEF_1_, C content was defined as 0.5^25^. As a result, forest stand scale C density (*C*d; MgC ha^−1^) is given as follows:

2 $$C{\rm{d}}=V{\rm{s}}\times {{\rm{BEF}}}_{1}\times 0.5.$$

BEF_2_ (unitless) is the conversion factor to expand stem biomass to the aboveground biomass (that is, stems, branches and leaves) and is presented for two age classes (below and above 20 years old) of 32 tree species, some alien species, other conifers and other broadleaf trees (see Table S1). *C*d using BEF_2_ can be written as follows:

3 $$C{\rm{d}}=V{\rm{s}}\times WD\times {{\rm{BEF}}}_{2}\times (1+R)\times \alpha ,$$

where *WD* is woody density (Mg m^−3^) and *R* (unitless) is the ratio of root biomass to aboveground biomass for a given tree species. Stem biomass ($V{\rm{s}}\times {WD}$) is converted into aboveground biomass by BEF_2_, then multiplied by ($1+R$) to calculate total biomass. *α* is C content: 0.51 and 0.48 for conifers and broadleaf trees, respectively^56^.

### Carbon stock estimation using m-NFI

The m-NFI-derived C stocks in 1961 and 1966 have been already reported in a previous study^25^; therefore, we estimated C stock for m-NFI in 2009–2013. Again, the numbers of actual forest and bamboo plots were 14,522 and 197, respectively. We calculated *C*d at 12,457 of the 14,522 forest plots using both BEF_1_ and BEF_2_, which were determined for the dominant tree species at the plot. We then obtained plot-averaged *C*d for each of the 11 forest types categorized in p-NFI. The total forest C stock was estimated as a total of each forest type C stock, which was calculated by multiplying the forest type averaged *C*d and forest type area for the 14,522 forest plots.

Surveyors could not reach or tree species could not be identified in 2,065 of the 14,522 forest plots. We assumed the 2,065 plots to be natural forests but could not apply a forest type (using the p-NFI categories). This means that while we could estimate forest type areas for 12,457 observation plots, those for 2,065 plots are unknown, which resulted an incorrect total forest type area in the 14,522 total forest plots (see Table S2).

Therefore, it is necessary to estimate the forest type areas for these 2,065 plots and the total for the 14,522 plots (Table S2). Assuming that the areas of each forest type in the p-NFI natural forest category are proportional to each forest type’s estimated stem volume in the natural forest category in the 2012 p-NFI, we assigned 11forest types to the 2,065 plots. The sum of the assigned plots for each forest type in the 2,065 non-observation (natural forest) plots and the 12,457 observation plots can be assumed to represent the area ratio of each forest type in the 14,522 total forest plots (Table S2). The forest area of each forest type can be calculated by multiplying Japan’s total forest area and this area ratio. Using aerial photographs, satellite images and field surveys, Japan’s total forest area was estimated to be 25.86 million ha (http://www.rinya.maff.go.jp/j/keikaku/tayouseichousa/tikuseki.html). The ratio of the bamboo forest plots to the actual forest plots (~1%) was subtracted from the total forest area, leaving 25.6 million ha as the total forest area.

### Carbon stock estimation using p-NFI

The p-NFI-estimated C stocks until 1995 have been already reported in previous studies^25,28^; therefore, we estimated C stock for the subsequent three p-NFIs in 2002, 2007 and 2012 using the same method as the previous studies. We calculated *V*s at plantations by dividing the p-NFI-estimated stem volume by the forest area for each factor, that is, prefecture, forest type and age class. Then, the *C*d was obtained by using the BEF_1_ calculation with the *V*s (see Eqs. 1 and 2). The C stocks at plantations were estimated by multiplying the *C*d and the forest area for each factor and summing the products.

No information is given in the p-NFI natural forest category about forest area; therefore, we estimated the C stock in natural forests without using the *V*s. We first obtained plantations’ BEF_1_ values for each prefecture and forest type and used these in place of those for natural forests. Then, the C stock in natural forests was estimated by multiplying the BEF_1_ and the total stem volume for each prefecture and forest type in natural forests and summing the products. Japan’s total forest C stock is determined by the sum of the C stocks in plantations and natural forests.

### Calculation of 95% confidence interval (CI)

We calculated the 95% CI for m-NFI-derived C stocks. No information on the C stock uncertainty was available in the previous study, which used the 1961 and 1966 m-NFIs to calculate the forest C stocks^25^. Thus, we assumed that the widths of the 95% CIs of the total forest C stocks calculated from the m-NFI of 1961 and 1966 were equal to that of total stem volume in the 1961 m-NFI, i.e., 5.4% of the mean value^57^. For m-NFI in 2009-2013, the 95% CI of the area-based C stock was calculated for each of the 11 forest types (Table S2) as follows:

4 $${\varepsilon }_{i}=\frac{{\sigma }_{i}}{\sqrt{{n}_{i}}}\times 1.96,$$

where ${\varepsilon }_{i}$, ${\sigma }_{i}$, and ${n}_{i}$ represent the half width of 95% CI (MgC ha^−1^), standard deviation (MgC ha^−1^), and sample size of area-based C stock of forest type *i*, respectively. Then the half width of 95% CI of total forest C stock (${\varepsilon }_{{\rm{T}}}$; MgC) can be calculated with the forest area of forest type *i* (${A}_{i}$; ha) as follows:

5 $${\varepsilon }_{{\rm{T}}}=\sqrt{\sum _{i}{({\varepsilon }_{i}\times {A}_{i})}^{2}}.$$

### Reproducing past carbon stocks of *Cr. japonica* and *Ch. obtusa*

Assuming that for a given forest type *i* (such as *Cr. japonica*) the net forest C uptake flux (*F*_*i*_; MgC ha^−1^ year^−1^) is proportional to the area-based C stock (*V*_*i*_/*A*_*i*_, in which *V*_*i*_ and *A*_*i*_ are C stock (MgC) and forest area (ha), respectively), the net C uptake of the forest type *i* (*F*_*i*_ × *A*_*i*_; MgC year^−1^) may be approximated as follows:

6 $${F}_{i}\times {A}_{i}=\frac{{F}_{{\rm{T}}}\times {V}_{i}}{{V}_{{\rm{T}}}},$$

where *F*_T_ and *V*_T_ are net total forest C uptake (MgC year^−1^) and total forest C stock (MgC), respectively. Thus, the past C stock of *i* at the year *Y*_1_ (*C*st_*Y*1, *i*_; MgC) can be calculated as:

7 $$C{{\rm{st}}}_{Y1,i}=C{{\rm{st}}}_{Y0,i}-{F}_{i}\times {A}_{i}\times ({Y}_{0}-{Y}_{1})=C{{\rm{st}}}_{Y0,i}-\frac{{F}_{{\rm{T}}}\times {V}_{i}}{{V}_{{\rm{T}}}}\times ({Y}_{0}-{Y}_{1}),$$

where *Y*_0_ is the reference year and *C*st_*Y*0, *i*_ is the C stock (MgC) at *Y*_0_.

## Supplementary information

Supplementary information.

## References

[1] Dixon R (1994). Carbon pools and flux of global forest ecosystems. Science.

[2] Bonan GB (2008). Forests and climate change: Forcings, feedbacks, and the climate benefits of forests. Science.

[3] Booth BBB (2012). High sensitivity of future global warming to land carbon cycle processes. Environ. Res. Lett..

[4] UNFCCC. Report of the Conference of the Parties on its seventh session, held at Marrakesh from 29 October to 10 November 2001. Addendum. Part two: Action taken by the Conference of the Parties. Volume I (UNFCCC, 2002).

[5] UNFCCC. Report of the Conference of the Parties serving as the meeting of the Parties to the Paris Agreement on the third part of its first session, held in Katowice from 2 to 15 December 2018. Addendum 1. Part two: Action taken by the Conference of the Parties serving as the meeting of the Parties to the Paris Agreement (UNFCCC, 2019).

[6] Kauppi PE (2010). Changing stock of biomass carbon in a boreal forest over 93 years. For. Ecol. Manage..

[7] Lawrence, M., McRoberts, R. E., Tomppo, E., Gschwantner, T. & Gabler, K. Comparisons of National Forest Inventories in National Forest Inventories: Pathways for Common Reporting (eds. Tomppo, E., Gschwantner, T., Lawrence, M. & McRoberts, R. E.) 19–32 (Springer Netherlands, 2010).

[8] Gschwantner T (2016). Comparison of methods used in European National Forest Inventories for the estimation of volume increment: towards harmonisation. Ann. For. Sci..

[9] Kauppi PE, Mielikainen K, Kuusela K (1992). Biomass and carbon budget of European forests, 1971 to 1990. Science.

[10] Goodale CL (2002). Forest carbon sinks in the Northern Hemisphere. Ecol. Appl..

[11] Shvidenko A, Nilsson S (2002). Dynamics of Russian forests and the carbon budget in 1961–1998: An assessment based on long-term forest inventory data. Clim. Change.

[12] Birdsey RA, Plantinga AJ, Heath LS (1993). Past and prospective carbon storage in United States forests. For. Ecol. Manage..

[13] Kurz WA, Apps MJ (1999). A 70-year retrospective analysis of carbon fluxes in the Canadian forest sector. Ecol. Appl..

[14] Fang J (2001). Changes in forest biomass carbon storage in China between 1949 and 1998. Science.

[15] Ciais P (2008). Carbon accumulation in European forests. Nat. Geosci..

[16] Pan Y (2011). A large and persistent Carbon sink in the World’s forests. Science.

[17] Eggers J, Lindner M, Zudin S, Zaehle S, Liski J (2008). Impact of changing wood demand, climate and land use on European forest resources and carbon stocks during the 21st century. Glob. Chang. Biol..

[18] Hu H, Wang S, Guo Z, Xu B, Fang J (2015). The stage-classified matrix models project a significant increase in biomass carbon stocks in China’s forests between 2005 and 2050. Sci. Rep..

[19] Wear DN, Coulston JW (2015). From sink to source: Regional variation in U.S. forest carbon futures. Sci. Rep..

[20] Hirata, Y., Matsumoto, M. & Iehara, T. Japanese national forest inventory and its spatial extension by remote sensing in Proceedings of the eight annual forest inventory and analysis symposium (eds. McRoberts, R. E., Reams, G. A., Van Deusen, P. C. & McWilliams, W. H.) 13–17 (U.S. Department of Agriculture, Forest Service, 2009).

[21] Matsushita K, Yoshida S (1998). Analysis of the recent situation and problems in forestry statistics in Japan. J. For. Econ..

[22] Kitahara F, Mizoue N, Yoshida S (2009). Evaluation of data quality in Japanese National Forest Inventory. Environ. Monit. Assess..

[23] Fukuda M, Iehara T, Matsumoto M (2003). Carbon stock estimates for sugi and hinoki forests in Japan. For. Ecol. Manage..

[24] Sasaki N, Kim S (2009). Biomass carbon sinks in Japanese forests: 1966–2012. Forestry.

[25] Fang J, Oikawa T, Kato T, Mo W, Wang Z (2005). Biomass carbon accumulation by Japan’s forests from 1947 to 1995. Global Biogeochem. Cycles.

[26] Wang Y (2011). Inventory-based estimation of aboveground net primary production in Japan’s forests from 1980 to 2005. Biogeosciences.

[27] Fang J (2014). Forest biomass carbon sinks in East Asia, with special reference to the relative contributions of forest expansion and forest growth. Glob. Chang. Biol..

[28] Fang J (2014). Evidence for environmentally enhanced forest growth. Proc. Natl. Acad. Sci..

[29] Japan Forestry Agency. Report of the accuracy verification survey for the 4th period forest ecosystem diversity survey in 2014. (in Japanese) (Japan Forestry Agency, 2015).

[30] Japan Forestry Agency. Report of the accuracy verification survey for the 4th period forest ecosystem diversity survey in 2018. (in Japanese) (Japan Forestry Agency, 2019).

[31] Kondo M, Saitoh TM, Sato H, Ichii K (2017). Comprehensive synthesis of spatial variability in carbon flux across monsoon Asian forests. Agric. For. Meteorol..

[32] Ito A (2008). The regional carbon budget of East Asia simulated with a terrestrial ecosystem model and validated using AsiaFlux data. Agric. For. Meteorol..

[33] Yamaji T (2008). Scaling-up technique for net ecosystem productivity of deciduous broadleaved forests in Japan using MODIS data. Ecol. Res..

[34] Japan Forestry Agency. Forest resources of Japan 2012. (in Japanese) (Japan Forestry Agency, 2012).

[35] Iwamoto, J. The development of Japanese Forestry in Forestry and the forest industry in Japan (ed. Iwai, Y.) 3–9 (University of Washington Press, 2002).

[36] Luyssaert S (2008). Old-growth forests as global carbon sinks. Nature.

[37] Stephenson NL (2014). Rate of tree carbon accumulation increases continuously with tree size. Nature.

[38] Nishizono, T., Sawata, S. & Awaya, Y. Structure and growth in an old-growth Sugi (Cryptomeria japonica) forest in Akita prefecture. *J. Jpn. For. Sci*. **88**, 8–14 (in Japanese with English abstract) (2006).

[39] Masaki, T. *et al*. How do thinning intensities affect long-term growth of tree height in a Japanese cedar plantation? *J. Jpn. For. Sci*. **95**, 227–233 (in Japanese with English abstract) (2013).

[40] Way DA, Oren R (2010). Differential responses to changes in growth temperature between trees from different functional groups and biomes: a review and synthesis of data. Tree Physiol..

[41] Chung H (2013). Experimental warming studies on tree species and forest ecosystems: a literature review. J. Plant Res..

[42] Nemani RR (2003). Climate-driven increases in global terrestrial net primary production from 1982 to 1999. Science.

[43] Kato T, Tang Y (2008). Spatial variability and major controlling factors of CO_2_ sink strength in Asian terrestrial ecosystems: evidence from eddy covariance data. Glob. Chang. Biol..

[44] Morino Y (2011). Temporal variations of nitrogen wet deposition across Japan from 1989 to 2008. J. Geophys. Res..

[45] Oren R (2001). Soil fertility limits carbon sequestration by forest ecosystems in a CO_2_-enriched atmosphere. Nature.

[46] Magnani F (2007). The human footprint in the carbon cycle of temperate and boreal forests. Nature.

[47] Norby RJ, Warren JM, Iversen CM, Medlyn BE, McMurtrie RE (2010). CO_2_ enhancement of forest productivity constrained by limited nitrogen availability. Proc. Natl. Acad. Sci..

[48] Wenzel S, Cox PM, Eyring V, Friedlingstein P (2016). Projected land photosynthesis constrained by changes in the seasonal cycle of atmospheric CO_2_. Nature.

[49] Japan Forestry Agency. Annual report on forest and forestry in Japan. (Japan Forestry Agency, 2018).

[50] Hoover C, Stout S (2007). The carbon consequences of thinning techniques: Stand structure makes a difference. J. For..

[51] Nilsen P, Strand LT (2008). Thinning intensity effects on carbon and nitrogen stores and fluxes in a Norway spruce (*Picea abies* (L.) Karst.) stand after 33 years. For. Ecol. Manage..

[52] Lin JC, Chiu CM, Lin YJ, Liu WY (2018). Thinning effects on biomass and carbon stock for young Taiwania plantations. Sci. Rep..

[53] Lehtonen A, Cienciala E, Tatarinov F, Mäkipää R (2007). Uncertainty estimation of biomass expansion factors for Norway spruce in the Czech Republic. Ann. For. Sci..

[54] Japan forestry Agency. Tables for calculating stem volume from diameter at breast height and tree height in eastern Japan. (in Japanese) (Japan forestry Agency, 1970).

[55] Japan forestry Agency. Tables for calculating stem volume from diameter at breast height and tree height in western Japan. (in Japanese) (Japan forestry Agency, 1970).

[56] Ministry of the Environment, Japan & Greenhouse Gas Inventory Office of Japan National greenhouse gas inventory report of Japan. p362(page6-14). (National Institute for Environmental Studies, 2018).

[57] Yoshida, S. Comparison between the past and present systems used in National Forest Inventory in Japan. *J. Jpn. For. Sci*. **90**, 283–290 (in Japanese with English abstract) (2008).

