# Supplementary material for: Carbon stock in Japanese forests has been greatly underestimated
Source: Sci Rep. 2020 May 12;10:7895. doi: 10.1038/s41598-020-64851-2 (PMC7217934; doi:10.1038/s41598-020-64851-2)
Supplement: Supplementary file 1 — Supplementary information. [file 41598_2020_64851_MOESM1_ESM.pdf]

Supplementary Information for

Carbon stock in Japanese forests has been greatly underestimated

Tomohiro Egusa[1,*], Tomo'omi Kumagai[1, 2] & Norihiko Shiraishi[1]

[1] Graduate School of Agricultural and Life Sciences, The University of Tokyo, 1-1-1 Yayoi, Bunkyo-ku, Tokyo 113-8657, Japan

[2] Institute for Space-Earth Environmental Research, Nagoya University, Furo-cho, Chikusa-ku, Nagoya 464-8601, Japan

[*] Corresponding author: Tomohiro Egusa (egusa@fr.a.u-tokyo.ac.jp)

Contents of this file

Tables S1 to S2

Table S1. Biomass expansion factor, BEF_2 (m^3 m^{-3}), proposed by Japan Forestry Agency.

Forest type	Number of plots	BEF_2 (age ≤ 20)	BEF_2 (age > 20)	R^1 (m^3 m^{-3})	D^2 (Mg m^{-3})
Abies firma	31	1.4	1.4	0.4	0.423
Abies sachalinensis	887	1.88	1.38	0.21	0.318
Acer	284	1.33	1.18	0.26	0.519
Alnus japonica	130	1.33	1.25	0.26	0.454
Betula	537	1.31	1.2	0.26	0.468
Castanea crenata	150	1.33	1.18	0.26	0.419
Cercidiphyllum japonicum	9	1.33	1.18	0.26	0.454
Chamaecyparis obtusa	1,639	1.55	1.24	0.26	0.407
Chamaecyparis pisifera	8	1.55	1.24	0.26	0.287
Cryptomeria japonica	2,758	1.57	1.23	0.25	0.314
Fagus	706	1.58	1.32	0.26	0.573
Ginkgo biloba	1	1.5	1.15	0.2	0.45
Kalopanax septemlobus	6	1.33	1.18	0.26	0.398
Larix kaempferi	543	1.5	1.15	0.29	0.404
Magnolia obovata	56	1.33	1.18	0.26	0.386
Paulownia tomentosa	1	1.33	1.18	0.26	0.234
Phellodendron amurense	11	1.33	1.18	0.26	0.344
Picea glehnii	102	2.17	1.67	0.21	0.362
Picea jezoensis	61	2.18	1.48	0.23	0.357
Pinus densiflora	683	1.63	1.23	0.26	0.451
Pinus thunbergii	25	1.39	1.36	0.34	0.464
Populus suaveolens	2	1.33	1.18	0.26	0.291
Quercus (ever green)	214	1.52	1.33	0.26	0.646
Quercus acutissima	62	1.36	1.32	0.26	0.668
Subgenesis *Quercus* (deciduous)	1,631	1.4	1.26	0.26	0.624
Thujopsis	46	2.38	1.41	0.2	0.412
Tilia japonica	161	1.33	1.18	0.26	0.369
Tsuga sieboldii	24	1.4	1.4	0.4	0.464
Ulmus	106	1.33	1.18	0.26	0.494
Zelkova serrata	61	1.58	1.28	0.26	0.611

Other conifers A	128	2.55	1.32	0.34	0.352
Other conifers B	4	1.39	1.36	0.34	0.464
Other conifers C	10	1.4	1.4	0.4	0.423
Other broad leaf A	310	1.37	1.37	0.26	0.469
Other broad leaf B	210	1.52	1.33	0.26	0.646
Other broad leaf C	860	1.4	1.26	0.26	0.624

[1]*R* represents the ratio of root volume to aboveground volume.

[2]*D* is biomass density.

Note.

For conifers: Other conifers A is applied to the following prefectures: Hokkaido, Aomori, Iwate, Miyagi, Akita, Yamagata, Fukushima, Tochigi, Gunma, Saitama, Niigata, Toyama, Yamanashi, Nagano, Gifu, and Shizuoka. Other conifers B is applied to Okinawa prefecture, and other conifers C is applied to the other prefectures.

For broad leaf trees: Other broad leaf A is applied to the following prefectures: Chiba, Tokyo, Kochi, Fukuoka, Nagasaki, Kagoshima and Okinawa. Other broad leaf B is applied to the following prefectures: Mie, Wakayama, Oita, Kumamoto, Miyazaki, and Saga. Other broad leaf C is applied to the other prefectures.

Table S2. Numbers of observation plots and the estimated non-observation plots, leading to the ratio of each forest type area to total forest area.

Forest type	Number of observation plots	Number of non-observation plots	Total number of plots	Area ratio (%)
Cryptomeria japonica	2758	7	2765	19.0
Chamaecyparis obtusa	1639	6	1645	11.3
Pinus	724	161	885	6.1
Larix	543	1	544	3.7
Abies and *Picea*	1117	102	1219	8.4
Fagus	706	218	924	6.4
Quercus	1810	558	2368	16.3
Betula	537	166	703	4.8
Other conifers	169	89	258	1.8
Evergreen broad leaf	674	208	882	6.1
Deciduous broad leaf	1780	549	2329	16.0
Total	12457	2065	14522	100.0
